# A Combinatorial Approach of High-Throughput Genomics and Mass Proteomics for Understanding the Regulation and Expression of Secondary Metabolite Production in Actinobacteria

**DOI:** 10.1128/mSystems.00862-21

**Published:** 2021-08-24

**Authors:** Vipin Gupta, Nirjara Singhvi

**Affiliations:** a The Ministry of Environment, Forest and Climate Change, New Delhi, Delhi, India; b PhiXgen Pvt. Ltd., Gurugram, India

**Keywords:** combinatorial biosynthesis, proteomics, *Actinobacteria*, secondary metabolite

## Abstract

Secondary metabolites produced by *Actinobacteria* are an important source of antibiotics, drugs, and antimicrobial peptides. However, the large genome size of actinobacteria with high gene coding density makes it difficult to understand the complex regulation of biosynthesis of such critically and economically important products. In the last few decades, apart from genomics sequences, high-throughput proteomics has proven beneficial to understand the key players regulating the expression pattern of secondary metabolite and antibiotic production in different experimental set-ups. In the past, we have been analyzing the genomics data and mass spectrometry-based proteomics to predict the regulation dynamics and crucial regulatory hubs in *Actinobacteria*. The multidirectional regulation and expression of the biosynthetic gene cluster responsible for the production of important metabolite take their cue from the other primary metabolism pathways with which they show intricate interactions in the interactome. The regulation occurs by not only the action and expression of the biosynthetic gene cluster but also the role of transcription factors and primary metabolic pathways. Using the key players of these interactomes, we can regulate the synthesis/production of these valuable peptides/metabolites. Simultaneously, the multi-omics approach has now opened new gateways in investigation, screening, and identification of naturally occurring antimicrobial peptides from actinobacteria which are beneficial for humans and also provide economic and industrial benefits to humankind.

## COMMENTARY

*Actinobacteria* represent one of the most varied Gram-positive microbial phyla with a high GC content and a remarkable range of complex morphologies, ranging from unicellular cocci to rods (*Micrococcus* and Mycobacterium, *Amycolatopsis*, *Frankia*, and *Streptomyces*) (1–3). Ecologically diversified, they have been reported from many varied habitats like terrestrial, aquatic, and mammalian microbiomes, etc. (4, 5). *Actinobacteria* are well known not only for their ability to produce antibiotics but also as a rich source of promising compounds with herbicidal, antitumor, antifungal, and anthelminthic activities. Common examples of these actinobacteria and their produced antibiotics are Streptomyces hygroscopicus (rapamycin [immunosuppressant]), Amycolatopsis mediterranei (rifamycin [antimicrobial]), Saccharopolyspora erythraea (erythromycin [broad-spectrum antibiotic]), Amycolatopsis orientalis (vancomycin [antibiotic]), etc. (6, 7). Due to such clinical properties, they are now acting as a major economic player in the pharmaceutical industry (8).

With the exponential increase in the global multifaceted phenomenon of multidrug resistance strains, there has been an alarmingly low rate of discovery of new antibiotics and their clinical approvals (9, 10). The apparent reason for the low rate of discovery for new antibiotics is likely due to the lack of deep understanding of the complex regulatory mechanism that is required to activate the expression of the biosynthetic gene clusters (BGCs) responsible for antibiotic synthesis in *Actinobacteria* under laboratory conditions (11). Genome sequencing projects have revealed that most of the natural-product-rich *Actinobacteria* have larger genomes (>8 Mb) that contain an average of ∼2,030 biosynthetic gene clusters (BGCs) for secondary metabolism. About half of these BGCs are nonribosomal peptide synthetases (NRPSs)/polyketide synthases (PKSs) (12). NRPSs/PKSs are basically the multidomain and multienzymatic megasynthase units that are involved in secondary metabolite synthesis in actinobacteria (13). In comparison to the other bacterial phyla like *Cyanobacteria*, where the gene coding density is around 52% (14), the gene coding density in *Actinobacteria* like *Corynebacterium* ranges up to 73% (15). This high coding density offers a wide scope of evolutionary dynamics in terms of horizontal gene transfer (16), gene duplication (17), gene decay (18), and genomic arrangements (19); thus, a prolonged genomic heterogeneity is associated with actinobacteria (15). Many groups have attempted to manipulate actinobacteria with a complex set of BGCs like Amycolatopsis mediterranei to augment the production of antibiotics but failed because of the lack of a stable cloning vector transformation system until 1991.

Later, the combinatorial biosynthesis approach (genetic engineering of natural biosynthetic clusters) used the information from genomic studies and established the first secondary metabolite databases like antiSMASH, which provides comprehensive information on BGC counts in actinobacteria (20). Combinatorial biosynthesis is considered the most successful approach of genetic engineering to produce new analogs of natural products by the process of modification of biosynthetic clusters and has been used worldwide for generating new antibiotics or their analogs. It was first used back in 1985, when Hopwood and group used this approach, targeting and cloning Streptomyces coelicolor BGC genes coding for actinorhodin into the medermycin and dihydrogranaticin antibiotic producers, respectively (21, 22). The new transformant thus generated produced large amounts of a new compound, including a hybrid antibiotic, mederrhodin A (with additional -OH group of actinorhodin). Similarly, a new compound-producing transformant was also generated producing dihydrogranatirhodin (23). Both these new analogs were found to harbor important clinical properties against bacterial infections (21–23).

A similar attempt was made by our group in 1991 with the successful development of the first hybrid plasmid vector construct as a prerequisite for transformation procedures for Amycolatopsis mediterranei, which further laid the foundation for its genetic manipulation (24). The cloning vector and transformation system were developed with the aim to study the biochemistry, physiology, and genetics of rifamycin B production in Amycolatopsis mediterranei. It was 2011 when the complete genome of *A. mediterranei* S699 was sequenced with 10.2 Mb and 9,575 predicted coding regions by the same group (12). Later, combinatorial biosynthesis was performed where substitution of the acyltransferase domain of module 6 of rifamycin polyketide synthase with that of module 2 of rapamycin generated the 24-desmethylrifamycin-B analog (9). This high-throughput genomics and combinatorial biosynthesis approach created a versatile platform for mining secondary metabolite-associated NRPSs/PKSs and their global regulatory genes in the other actinobacteria like *Streptomyces* and *Actinoplanes*, *Nocardia*, and *Sorangium* spp. for production of bioactive molecules (25, 26). Thus, basic understanding of the biosynthesis of antibiotics like erythromycin, rapamycin, and rifamycin opened up new possibilities for developing new molecules by their genetic manipulations (27–30). This method was found more convenient than all the other chemical and biological modifications of existing antibiotic molecules (29, 31–33). But with time, the number of successful attempts started to drop, and no new analogs were produced. The major problem faced by scientists across the world was the reduction in the amount of secondary metabolite production in the mutants compared to the wild-type strains after their genetic manipulations (9). Apart from the complicated handling conditions for the actinobacteria, their complex genomic architecture also heightens the failure in genetic manipulations. It was seen that the manipulations may result in changes in the expression profile of the gene involved in the antibiotic production pathway (10). The major reason found was the changed structure of the product, which creates impact on the functioning in downstream processing in the cell or disruption of the closely linked gene during the manipulation process (10), thus resulting in higher probabilities of having low potent bioactivity.

In order to overcome these limitations of the genomic and combinatorial biosynthesis approach, the proteomics approach became popular. Initial proteomic investigations dealing with microbial secondary metabolism were largely based on targeted proteomic profiling formats and included two approaches for NRPS/PKS study: PrISM (PRediction Informatics for Secondary Metabolomes) and OASIS (Orthogonal Active Site Identification System) (34, 35). The two used different methods for the targeting or enrichment of PKS and NRPS. PrISM is a computational biology approach that selects nonribosomal peptides and type I and II polyketides by their large sizes (35). OASIS (experimental approach) chemically reacts with the active sites of NRPS/PKS for affinity enrichment and offers a valuable tool for enzyme discovery, culture condition optimization, and strain comparison (34). However, a gap still remained in genetics-based investigations of polyketide synthase (PKS) and nonribosomal peptide synthetase (NRPS) biosynthetic pathways and understanding of their regulation, interaction, and activity in living systems. To overcome these hurdles, our group attempted strain-specific high-throughput proteomic mining overlapped with genomics using interactomics (36, 37). This multi-omic approach was employed to improve the production of 24-desmethylrifamycin-B, which was highly effective against rifampin-resistant strains of the tuberculosis bacterium (RR-TB) but had low yield. Where the proteomics approach unveiled the expression profile during the rifamycin production at a different timescale and was a major cause of low yield, the interactomics approach helped to uncover the major regulatory hub proteins that were regulating the secondary metabolite production (10, 36). These hubs were later targeted to enhance the production of the antibiotic.

Thus, applying a similar approach and combining the proteomics and *in silico* interactomics approaches, the unknown mechanism can be understood better and the information can be then used for promoting antibiotic yield. We strongly believe that overlapping high-throughput genomics with proteomics and their integration via interactomics can crucially provide the upper hand for understanding the current gaps in the regulatory structures of actinobacteria.
